# Comparative analysis of RNA expression in a single institution cohort of pediatric cancer patients

**DOI:** 10.1038/s41698-025-00852-6

**Published:** 2025-03-22

**Authors:** Yvonne A. Vasquez, Holly C. Beale, Lauren Sanders, A. Geoffrey Lyle, Ellen T. Kephart, Katrina Learned, Drew Thompson, Jennifer Peralez, Amy Li, Min Huang, Kimberly A. Pyke-Grimm, Sofie R. Salama, David Haussler, Isabel Bjork, L. Spunt Sheri, Olena M. Vaske

**Affiliations:** 1 Department of Molecular, Cell and Developmental Biology, University of California, Santa Cruz, CA USA; 2 UC Santa Cruz Genomics Institute, Santa Cruz, CA USA; 3 Department of Biomolecular Engineering, School of Engineering, University of California, Santa Cruz, CA USA; 4 Stanford University School of Medicine, Stanford, CA USA; 5 Department of Nursing Research and Evidence-Based Practice at Stanford Medicine Children’s Health, Stanford, USA; 6 Present Address: Blue Marble Space Institute of Science, NASA Ames GeneLab, Silicon Valley, CA USA; 7 Present Address: Foundation to Advance Vascular Cures, Redwood City, CA USA

**Keywords:** Cancer, Computational biology and bioinformatics

## Abstract

With the low incidence of mutations in pediatric cancers, alternate genomic approaches are needed to identify therapeutic targets. Our study, the Comparative Analysis of RNA Expression to Improve Pediatric and Young Adult Cancer Treatment, was conducted by the UC Santa Cruz Treehouse Childhood Cancer Initiative and Stanford University School of Medicine. RNA sequencing data from 33 children and young adults with a relapsed, refractory or rare cancer underwent CARE analysis to reveal activated cancer driver pathways and nominate treatments. We compare our pipeline to other gene expression outlier detection approaches and discuss challenges for clinical implementation. Of our 33 patients, 31 (94%) had findings of potential clinical significance. Findings were implemented in 5 patients, 3 of which had defined clinical benefit. We demonstrate that comparator cohort composition determines which outliers are detected. This study highlights the clinical utility and challenges of implementing comparative RNA sequencing analysis in the clinic.

## Introduction

Although overall clinical outcomes for pediatric cancer patients have improved over the past few decades, patients with recurrent/refractory or rare tumors still fare poorly^1^. DNA-mutation-guided therapies have improved outcomes for some pediatric cancers^2^. However, mutation analysis alone is often insufficient to identify therapeutic targets in most pediatric cancers because of the low incidence of clinically actionable mutations^3,4^. This emphasizes the need for alternate genomic approaches to identify additional treatment biomarkers and therapeutic targets.

Several studies have demonstrated the utility of RNA sequencing (RNA-Seq) combined with DNA mutation analysis for pediatric cancer patients, primarily to identify clinically actionable variants^5,6^ or fusion transcripts^2,7–13^. Studies in adult cancers^14^ have begun to look at abnormal gene expression to identify overexpressed pathways and targets for treatment. However, this approach is underexplored in pediatric cancers because the interpretation of gene expression results requires either matched normal tissues or comparator cohorts, which are harder to obtain for pediatric tumors^15^. Additionally, interpretation and integration of genomic data into clinical care, requires a close partnership of multiple professionals, which can be challenging due to funding constraints, regulatory barriers and limited interoperability of medical systems.

Several multi-tumor-type pediatric cancer precision medicine studies^6,16–18^ have utilized RNA-Seq-derived gene expression to identify druggable genes and pathways that are highly expressed in patient tumor samples (termed gene expression outliers). However, they utilized inconsistent methods to identify such outliers, leading to difficulties in comparing across studies. The main inconsistency in identifying gene expression outliers is the choice of comparator cohort to assess abnormal gene expression in a patient’s tumor sample. For instance, Zero Childhood Cancer^17^ and INFORM^16^ utilized all other patients in the study as comparator cohorts to define gene expression outliers, while the Personalized Onco-Genomics (POG)^18^ Program utilized The Cancer Genome Atlas (TCGA) tumor and normal datasets^19^.

We previously described Comparative Analysis of RNA Expression (CARE), a comparative RNA-Seq approach for identifying overexpressed genes and pathways in pediatric tumors^20^. This approach relies on large shared genomic datasets consistently processed for combined analysis. Unlike most other implementations of outlier detection, CARE compares expression in the focus RNA-Seq sample to multiple cancer cohorts. In case studies, we have demonstrated the clinical utility of the CARE approach for both identifying treatments^21,22^ and for refining diagnoses of rare tumors^23^. Here, we evaluate our approach in a cohort of 33 pediatric, adolescent, and young adult patients (age at diagnosis <30 years) with recurrent/refractory or rare cancer treated at a single institution (Supplementary Data 1). We demonstrate that comparative RNA-Seq analysis paired with DNA mutation analysis was potentially informative for most study participants, including three patients who received the identified therapy and derived clinical benefit. We explore the impact of comparator cohort composition on gene expression outlier analysis and highlight the importance of both cohort size and composition. This study underscores the potential added value of gene expression profiling in pediatric oncology and highlights their unique challenges.

## Results

### Patient characteristics

Thirty-three eligible patients were enrolled in CARE IMPACT between March 2018 and August 2020 (Supplementary Data 1); 32 patients had a recurrent/refractory tumor, and one had a newly diagnosed high-risk cancer without an established standard of care. The median age at diagnosis was 11 years (range 0–24 years); 55% were male. Soft-tissue sarcoma was the most frequent cancer subtype (*n* = 16, 48%). The median time from collection of a patient tissue sample to submission of the CARE IMPACT report to the treating oncologist was 20 days (range 8–38 days, Supplementary Data 2). Overall turnaround times remained consistent throughout the study regardless of disease type.

### CARE IMPACT findings

CARE IMPACT analysis was applied to compare each patient’s tumor RNA-Seq dataset against various comparative cohorts to identify druggable gene overexpression outliers, expressed mutations, expressed fusions, and other highly expressed genes. Automated CARE findings included overexpression outliers identified from the CARE pan-cancer and pan-disease analyses. Findings categorized as “generated using human curation” are those identified using curated similar disease cohorts; those present in only one of four pan-disease cohorts; highly expressed non-outliers implicated by mutation; and mutations and fusion genes.

**Fig. 1:**
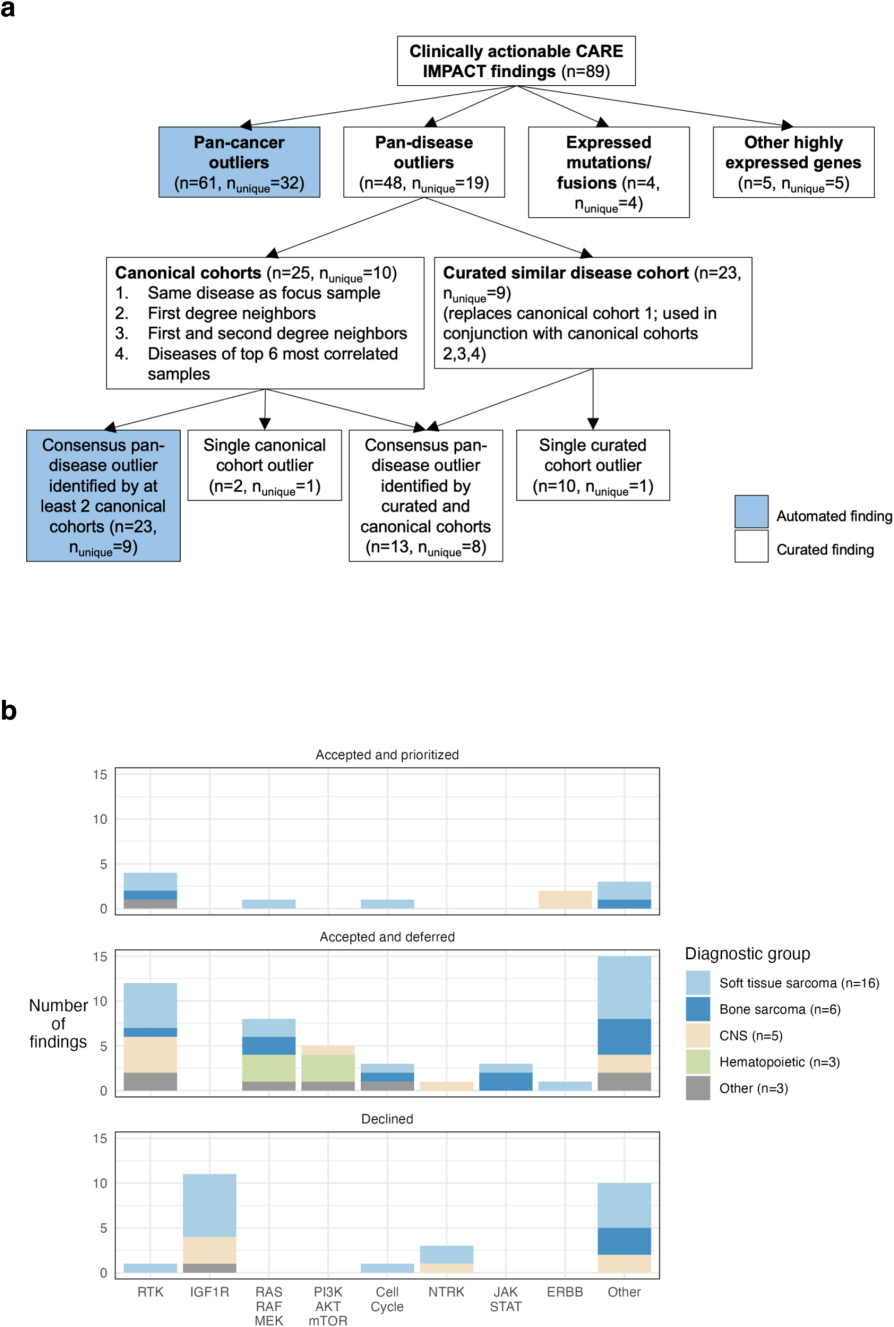
CARE IMPACT findings summarized. **a** Breakdown of 89 clinically relevant CARE IMPACT findings by method of detection. The automated CARE IMPACT pipeline and human curation identified 89 findings with evidence from four possible sources: pan-cancer outliers, pan-disease outliers, expressed mutations/fusions, or other highly expressed genes. A fraction of the findings were identified by both the pan-cancer and pan-disease analyses. The total number of RNA-Seq findings that fell into each category are enumerated in parentheses for each finding type. Findings that were uniquely identified by only the method listed in the box are designated by n_unique_. Pan-disease findings are further categorized by the cohorts used and the number of pan-disease cohorts used to detect them. Consensus outliers were defined as those identified by at least two cohorts. **b** Breakdown of 89 CARE IMPACT findings by tumor vulnerability category and prioritization status for 33 patients studied. Tumor vulnerability category is defined as the category a gene falls under in terms of its function. Each bar graph represents a prioritization status designated by a clinician. The bars show total counts of CARE IMPACT findings in each tumor vulnerability category, colored by diagnostic group. The number of patients in each diagnostic group is indicated in the legend.

CARE IMPACT analysis of the 35 tumors identified 89 clinically relevant findings presented in clinical genomics tumor boards (Supplementary Data 3). Of these findings, 32 (36%) were uniquely identified by the automated pan-cancer pipeline, 9 (10%) were uniquely identified by the automated pan-disease pipeline (canonical consensus), 8 (9%) findings were uniquely identified by the automated pan-disease pipeline after adding a curated pan-disease cohort (curated consensus), 11 (12%) findings were uniquely identified by other means involving curation (mutations, fusions, other highly expressed genes, single cohort pan-disease outliers), and 29 (33%) findings were identified by both pan-cancer and pan-disease pipelines (Fig. 1a and Supplementary Data 3).

Of the 89 CARE IMPACT findings identified and reported to Stanford, 70 (79%) were identified by our automated CARE pipelines, and 19 (21%) were identified only by human curation. Curation identified additional findings for 13 patients, three of whom had no findings identified by the automated pipeline.

All patients had at least one clinically relevant CARE IMPACT finding identified (Supplementary Data 3, Fig. 1b). While in over 10 cases, IGF1R was identified as a druggable gene expression outlier; it was not considered a useful finding because of the broad failure of IGF1R inhibitors in clinical trials^24^. In contrast, gene expression outliers in the other vulnerability categories, notably Receptor Tyrosine Kinases (RTKs), were considered useful because of the availability of clinical data on the inhibitors.

### CARE IMPACT treatment outcomes

For CARE IMPACT findings nominated for each patient, we assessed the clinical relevance, how the patient’s care was affected, and response to therapy for those who received treatment (Supplementary Fig. 1, Supplementary Data 3). CARE IMPACT findings provided helpful information for selecting treatment in five cases (Table 1). Three patients had a response of stable disease after two months of treatment that was maintained for at least six months, and two had disease progression (Table 1, Supplementary Fig. 1). One of these patients diagnosed with myoepithelial carcinoma^25^ (TH34_1352), a rare malignant tumor, was ultimately rendered disease-free with an identified therapy and surgery.

**Table 1:** Patients treated based on CARE IMPACT findings

Patient ID	Age at diagnosis (years)	Disease	Gene target	Finding type	Pan-disease type	Cohort type	Drug given	Therapy response (duration)	Current status after treatment discontinuation (duration)
TH34_1349	11	Gastrointestinal stromal tumor	KIT	pan-cancer, pan-disease	single cohort	curated	sunitinib	SD (37 mo); therapy discontinued due to insurance issues. PD	AWD (1.9 yrs)
TH34_1352	1.9	Myoepithelial carcinoma	FGFR1, FGFR2, PDGFRA	pan-cancer, pan-disease	single cohort	canonical	pazopanib	PD	NED (2.2 yrs)
			CCND2	pan-disease	single cohort	canonical	ribociclib	SD (12 mo), followed by complete resection of residual tumor and 12 additional months of the same therapy	
TH34_1381	2.5	Ependymoma	ERBB2	pan-disease	consensus	canonical	neratinib	PD	DOD
TH34_1456	12	Osteosarcoma	KDR/VEGFR2	pan-cancer, pan-disease	consensus	canonical	cabozantinib	SD (11 mo), then PD	DOD
TH34_2351	5	Embryonal rhabdomyosarcoma	MAP2K1	95th percentile			trametinib	PD	DOD

Five patients were treated based on CARE IMPACT findings. The response to therapy and the status of the patients after treatment are described. Therapy response is specified as stable disease (SD) or progressive disease (PD). The patient’s status after treatment is specified as no evidence of disease (NED), alive with disease (AWD), or dead of disease (DOD).

In five additional cases, the clinician was interested in administering a treatment supported by the CARE IMPACT analysis, but it was ultimately not used due to rapid disease progression (*n* = 3), unavailability of the therapy (*n* = 1), or because the family requested therapy initiation before an investigational new drug (IND) application could be completed (*n* = 1) (Supplementary Fig. 1).

In 21 cases, the CARE IMPACT findings deemed as most helpful information by the clinician were deferred because the treating oncologist elected a treatment option with more published evidence of efficacy (Supplementary Fig. 1). In 16 cases, the primary reason for deferring the identified treatment was the existence of another treatment with more published data in the specific condition (Supplementary Results). In three cases, the CARE IMPACT findings were deferred because the patient had not yet received the known standard of care treatment for their disease. In these cases, the clinician indicated they would consider using the identified treatment if the standard therapy was ineffective. The patient no longer needed treatment in two cases.

For two patients, the clinician did not find any of the nominated CARE IMPACT findings informative for treatment. In one case, the clinician was aware of studies in the cancer being treated showing limited efficacy of the drug identified in the CARE IMPACT analysis. In another case, all findings lacked FDA-approval.

Human curation identified informative findings for which the patient received therapy in three cases. Of those three patients, one achieved stable disease, one achieved no evidence of disease, and one had progressive disease on the CARE IMPACT elected therapy (Table 1, “single cohort” pan-disease type).

### Comparison of outliers detected by alternate comparator cohorts vs CARE analysis

While other pediatric precision medicine studies^6^ have utilized gene expression outlier analysis to identify targets for therapy, there is no consistency in how the outliers are defined in terms of the composition of comparator cohorts. To evaluate the impact of cohort composition on the outlier results, we compared the outliers detected by CARE to outliers detected using other common outlier detection strategies (e.g. single study cohort^16^, TCGA cohort^18^, a diversity of pediatric and young adult tumors^17^).

The automated CARE analysis identified 89 clinically relevant outliers (Supplementary Fig. 2, Supplementary Data 4; summarized in Table 2). Seventy-two percent of the outliers had pathway support (Table 2). Outliers were detected in 33 of the 35 analyzed tumor datasets and 31 of the 33 patients. Of the three alternative cohorts used for outlier detection, comparisons to the TCGA cohort best replicated the automated CARE results, identifying 82% of the outliers, followed by a single institution cohort (Stanford) (43%) and the pediatric cohort (22%). Most of the automated CARE outliers detected by comparison to the Stanford cohort or the pediatric cohort were also detected by comparison to TCGA (Supplementary Fig. 3).

**Table 2:** Pathway support status of outliers detected by different comparative cohorts

Comparison cohort	Number of CARE druggable outliers detected	Number of CARE druggable outliers with pathway support	Fraction of CARE druggable outliers with pathway support
Treehouse CARE total	89	64	64/89 (72%)
Treehouse CARE pan-cancer only	72	47	47/72 (65%)
Treehouse CARE pan-disease only	38	29	29/38 (76%)
Stanford	38	25	25/38 (66%)
TCGA	73	53	53/73 (73%)
Pediatric	20	10	10/20 (50%)

### TCGA-only cohort is of limited use for outlier analysis of pediatric samples

**Fig. 2:**
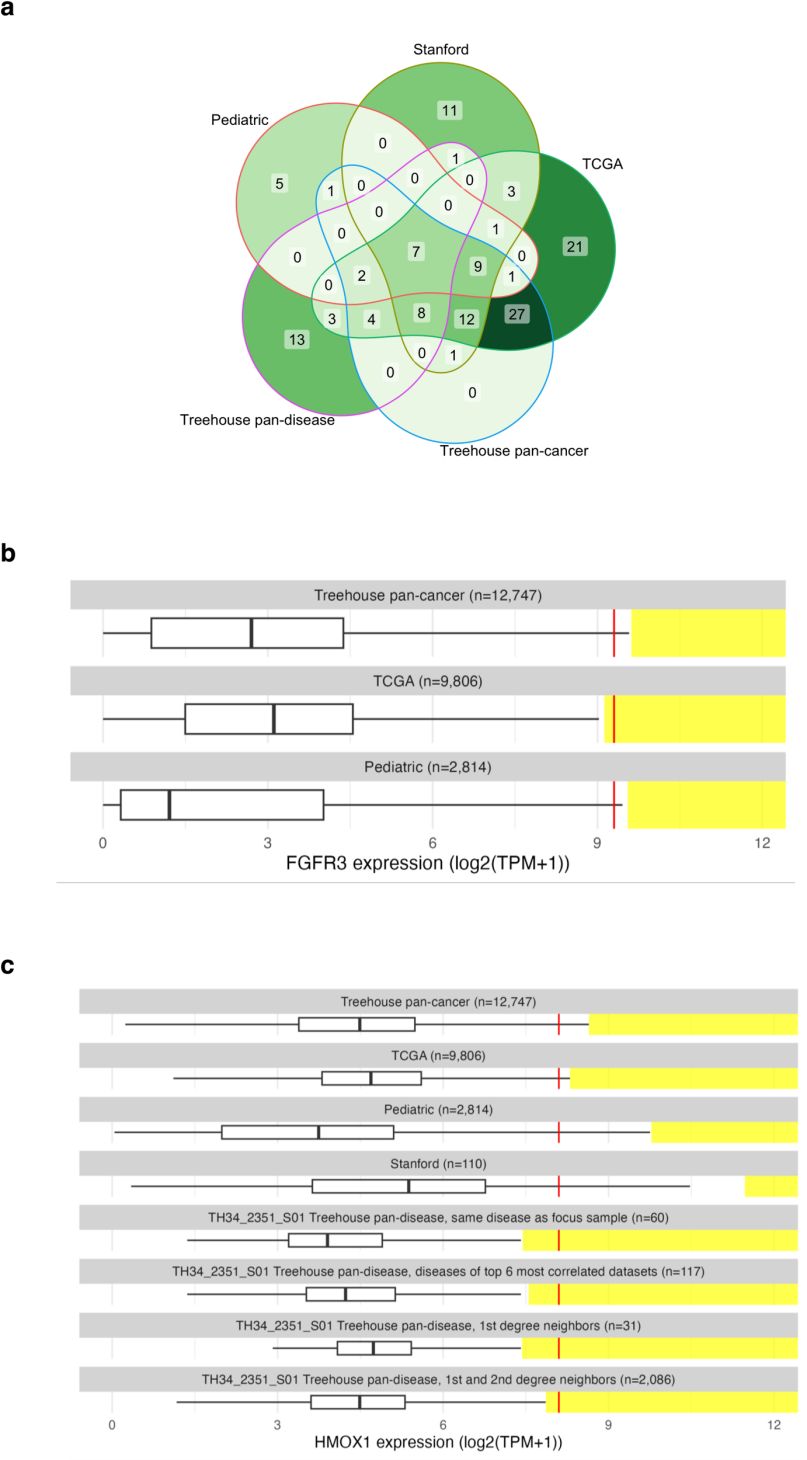
The choice of comparator cohort affects the outlier status. **a** Numbers of outliers detected relative to the comparator cohort(s) used. The total number of outliers detected relative to each combination of cohorts is displayed in each intersecting region. The largest set consists of 27 outliers detected relative to both the Treehouse pan-cancer cohort and TCGA. **b** FGFR3 expression in the patient sample TH34_1455_S01 illustrates the impact of cohort selection on outlier status. The FGFR3 expression level in the sample is denoted with a vertical red line plotted with respect to the distribution of FGFR3 gene expression in log2(TPM + 1) across the comparator cohort (x-axis). The outlier range is denoted with a yellow bar. **c** HMOX1 expression level in the sample TH34_2351_S01 (red) relative to various comparator cohorts. Vertical red line denotes gene expression level in the sample with respect to the distribution of HMOX1 gene expression in log2(TPM + 1) across the comparator cohort (x-axis). The outlier range is denoted with a yellow bar.

We next considered outliers detected solely by approaches other than the automated CARE analysis (Fig. 2a). Twenty-five of these outliers were detected using outlier analysis against the TCGA cohort, and most outliers (21/25, 84%) were uniquely detected in this comparison and not present in comparisons against the Stanford or pediatric cohorts. To address why using our TCGA comparative cohort yielded outlier findings not identified by other cohorts, we analyzed the identity of these genes with outlier expression and how their outlier thresholds compared to those set by other comparative cohorts.

Of the 16 genes in which these 21 outliers were detected, all 16 have wider distributions of expression in pediatric cohorts compared to TCGA, leading to higher outlier thresholds in cohorts with pediatric datasets. For example, the *FGFR3* IQR is 3.69 log2(TPM + 1) in the pediatric cohort and 3.05 in the TCGA cohort (Fig. 2b). Even though the median *FGFR3* expression value is lower in the pediatric cohort than in the TCGA cohort (Supplementary Data 5), the outlier threshold is higher due to the larger IQR. If we had used the TCGA cohort instead of the Treehouse compendia as the comparison cohorts in the CARE IMPACT study, we would have identified 98 gene expression outliers, 21 (21%) of which would not be relevant to pediatric cancers, as their outlier status would not be replicated if pediatric datasets were added to the comparator cohorts (Fig. 2b).

### Using sample-specific comparator cohorts increases findings by 11%

The CARE pan-disease analysis uses a personalized comparator cohort customized to the transcriptome of a patient’s tumor. The personalized cohorts that are generated for each patient’s tumor include 1) datasets from tumors with the same diagnosis as the focus sample, 2) molecularly similar RNA-Seq datasets (first degree neighbors), 3) first and second degree neighbors (first degree neighbors plus RNA-Seq datasets molecularly similar to them), and 4) datasets from diseases present among the top 6 most correlated datasets. To assess the added value of including sample-specific comparator cohorts in our outlier analysis pipeline, we quantified how many Treehouse findings were uniquely identified by Treehouse pan-disease analysis but missed by predefined CARE cohorts.

In total, Treehouse pan-disease analysis added 13 findings not detected by predefined cohorts, increasing the total number of findings by 11% from 117 to 130 (Fig. 2c). For example, *HMOX1* in TH34_2351_S01 was not identified as an outlier gene in any of the static cohorts (Treehouse compendium, TCGA, pediatric and Stanford cohorts; Fig. 2c). However, *HMOX1* is expressed exceptionally highly compared to the personalized cohorts that are generated for this dataset: other embryonal rhabdomyosarcomas (“same disease”), transcriptionally correlated datasets (“First degree neighbors” and “First and second degree neighbors”), and datasets with the same disease as one of the top 6 most correlated datasets.

### Adding more datasets to comparator cohorts improves specificity

To assess how size and composition of comparator cohorts impact outlier results, we compared the results of CARE analysis using the version of the Treehouse compendium at the initial time of the patient’s tumor analysis and using the most up do date compendium version v11.

As part of our standard process, our data compendia are updated regularly with new datasets. We also routinely perform quality control analysis, including review of the annotations that accompany datasets, and investigations for possible batch effects. We discovered annotation errors affecting 20 of the 27 ependymomas in version 8; these datasets were removed in the subsequent versions of the compendium. By compendium version v11, we had also added 95 high quality ependymoma datasets from five studies. Consequently, while *ERBB2* was detected as a pan-disease outlier by CARE when the TH34_1381_S01 dataset was analyzed using the Treehouse compendium version 8 (v8), this outlier was not detected against the updated compendium version 11 (v11).

The datasets most highly correlated to TH34_1381_S01 in the v8 compendium included ependymoma, glioblastoma multiforme and glioma. All correlations were above our required threshold^20^ of 0.875 for that compendium, with the highest correlation being 0.90. The “diseases of the top most correlated datasets” comparator cohort consisted of datasets originating from the three diseases, and TH34_1381_S01’s *ERBB2* expression exceeded the outlier threshold. It also exceeded the threshold for the “1st and 2nd degree correlated samples” cohort, making it a consensus pan-disease outlier. The patient was treated with an *ERBB2* inhibitor on a clinical trial but experienced disease progression.

In contrast, all of the most correlated datasets identified in the v11 compendium were ependymoma, and the lowest correlated was 0.92. Consequently, the “diseases of the top most correlated datasets” comparator cohort in v11 included only ependymomas. TH34_1381_S01’s *ERBB2* expression did not exceed the outlier threshold of the cohort. The new ependymomas indicated that TH34_1381_S01’s *ERBB2* expression was not exceptional for ependymomas. Although the expression also exceeded the threshold for the “1st and 2nd-degree correlated samples” cohort, it was not a consensus pan-disease outlier and was not reported in Table 1 or Fig. 2. This case illustrates the importance of increasing the size of the comparator cohort with high-quality datasets.

## Discussion

The mutational burden of childhood cancers is much lower than that of adult cancers, resulting in a lower frequency of molecular targets for therapy^11^. In this cohort of 33 recurrent, refractory or rare pediatric tumors, implementation of RNA-Seq-based gene expression analysis alongside DNA mutation analysis in a clinical setting was feasible and produced informative molecular abnormalities in all patients. Of our 33 patients, 31 (94%) had CARE IMPACT findings of potential clinical significance. These findings were implemented in 5 patients, and in 3 out of 5, the treatments produced defined clinical benefit.

In addition to identifying novel druggable aberrations, comparative RNA-Seq can clarify which patients may benefit from a biomarker-targeted therapy in the absence of the established biomarker. For example, our patient with GIST (TH34_1349) and wild-type *KIT* in the setting of germline *SDHC* mutation had *KIT* overexpression and benefitted from sunitinib treatment. Even though sunitinib is a known treatment strategy for wild-type GIST, this is a unique case since most GISTs with wild type *KIT* do not respond to tyrosine kinase inhibitor (TKI) treatment^26^. While it has been previously reported that *KIT* overexpression can be a mechanism of resistance to imatinib^27^, this observation was made in a setting of mutant *KIT*. It has also been reported that the mechanisms of wild type and mutant *KIT* overexpression are distinct, and so the expression level of *KIT* should be considered differently in the KIT-mutant and KIT-wild type disease. Therefore, *KIT* overexpression may serve as a biomarker of response to TKIs in the setting of wild-type *KIT* where KIT-targeted therapy might not otherwise be prioritized. However, more data on the relationship of *KIT* overexpression and response to TKI in the setting of wild type *KIT* should be collected to assess whether the amount of wild type *KIT* transcript in a tumor could be utilized to predict the patient’s response to imatinib and other TKIs.

Despite most patients harboring an informative finding, these findings were only implemented in five cases, and the suggested treatments were often deferred in favor of other therapies. This was because Treehouse CARE is not a clinically validated assay, and we could not conduct a formal clinical trial in which therapies suggested by CARE would have to be implemented. The paucity of clinically validated RNA-Seq assays and the availability of multiple treatment options and clinical trials available highlight the challenge of evaluating the impact of treatments guided by research genomics assays. For 19 of 33 (58%) of the patients with CARE IMPACT findings, other treatments with more data in the disease (including standard of care) were available for consideration by the clinical teams. Proving the value of genomics-guided treatments will depend on doing studies where patients receive the identified therapy. Clinical validation of this comparative RNA-Seq protocol, which is underway, will aid in further evaluation of the clinical utility of this approach in patient care.

Most tumors were analyzed after the standard of care treatments had been exhausted, leaving the patients prone to rapid clinical decline. For three patients in which RNA-Seq analysis identified a treatment that would have been implemented, the patients had rapid disease progression and died before they could receive the treatment. This emphasizes the need for timely integration of molecular analysis in cancer care.

Another challenge to implementing personalized genomic approaches in clinical practice is the availability of drugs. Here we restricted our analysis to FDA-approved drugs because they can be used off-label. However, for one patient, an investigational NOTCH inhibitor was requested under compassionate use during palliative RT for spinal cord compression. Oral etoposide was started instead because the family was anxious to start systemic therapy and did not want to await FDA approval of the IND.

A key challenge in the clinical implementation of RNA-Seq-based gene expression is standardizing gene expression outlier analysis. While there are inherent limitations to doing any comparative analysis in rare diseases like pediatric cancer, we demonstrate that the composition of comparator cohorts determines which outliers are detected and that large and diverse cohorts containing data from tumors similar to the patient’s produce the most clinically relevant outlier results. Comparing pediatric datasets to TCGA-only cohorts produces gene expression outliers with limited relevance to pediatric cancers, i.e. the identification of gene expression that is exceptionally high for adult cancers but not for pediatric cancers. Approximately one-fifth of the outliers detected when comparing to TCGA only (which is >96% adults) are not detected in comparison to the other cohorts, which contain a minimum of 22% pediatric datasets, indicating that these outliers are due to the paucity of pediatric samples in the TCGA cohorts.

In addition, we show that our pan-disease analysis, which compares a dataset to dynamically generated, patient-specific cohorts based on disease and molecular similarities, generates orthogonal results. Of the 38 pan-disease findings, 34.2% were not detected by any predefined single-cohort analysis. Pan-disease findings identify how the patient’s tumor differs from other similar tumors, which may highlight potential therapeutic alternatives for patients whose disease does not respond to the standard-of-care treatment. Therefore, an ideal comparator cohort would be composed of hundreds of datasets of each pediatric and adult tumor type. Important limitations to constructing large comparator cohorts for gene expression outlier analysis are the siloing of RNA-Seq data and the differences in the processing and analysis of RNA-Seq datasets, hindering the merging of multiple datasets^28,29^. We anticipate that NCI’s Childhood Cancer Data Initiative (CCDI) will help solve the data siloing dilemma by creating a federated framework in which RNA-Seq data could be shared across stakeholders.

This study is limited by a relatively small cohort of heterogeneous pediatric diseases. Further examinations of clinical utility of comparative RNA-Seq in larger cohorts of single diseases are warranted.

The incorporation of RNA-Seq-based expression analysis to identify clinically relevant therapeutic targets in difficult-to-treat pediatric tumors is feasible as a collaborative effort of an interdisciplinary team. This approach revealed druggable aberrations in most of our cohort and can be performed within the time frame required for patient care. In all cases, we convened an interdisciplinary, interactive genomic tumor board tailored to a specific patient’s needs. This tumor board was highly educational to both clinicians and researchers and led to improvements in the analysis and reporting process (discussed in a separate manuscript). Therefore, we believe that close partnerships of multiple professionals are essential to a successful precision medicine program.

## Methods

### Study design and patients

**Fig. 3:**
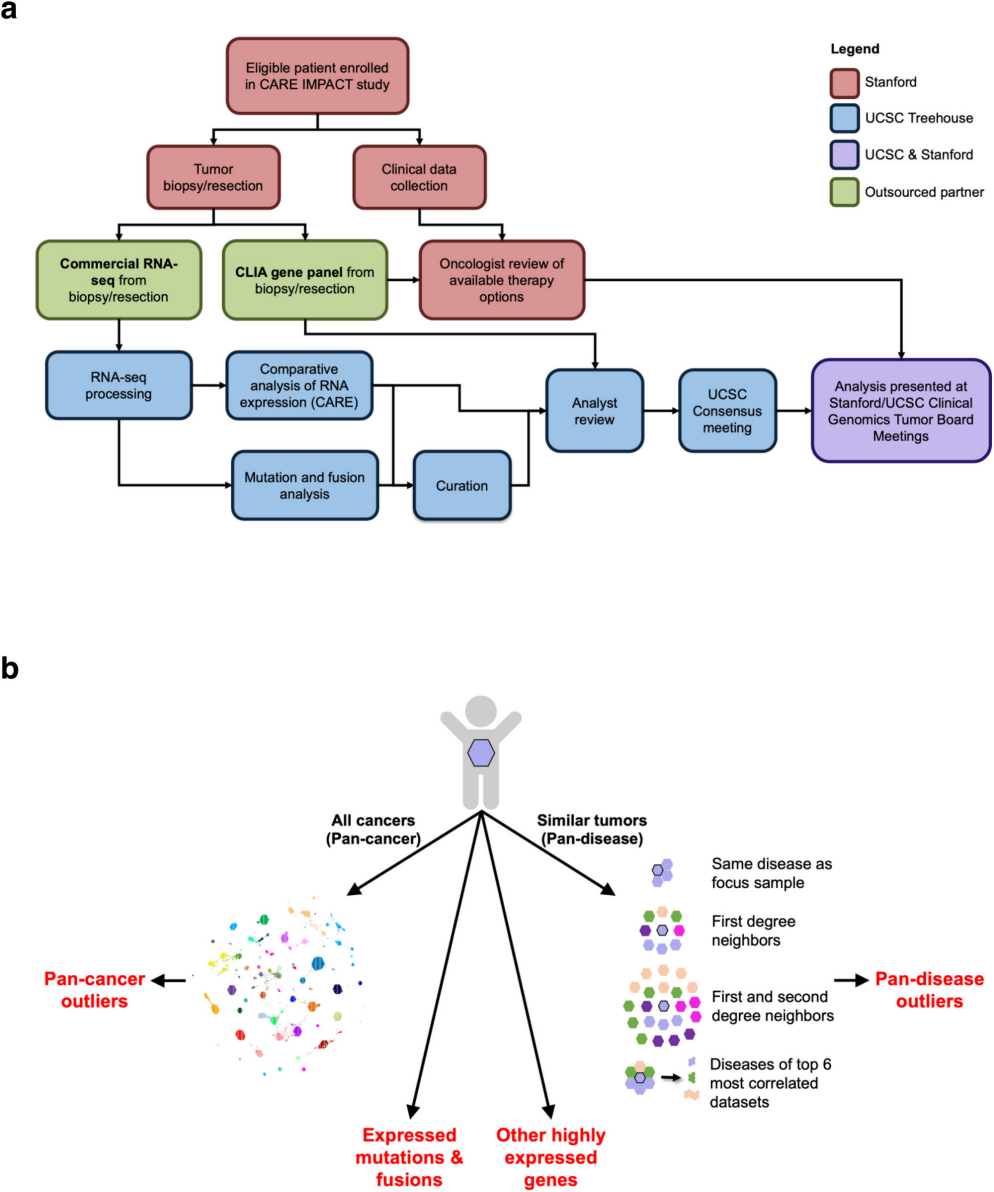
CARE IMPACT study workflow. **a** The CLIA gene panel ordered was either a Foundation Medicine gene panel (https://www.foundationmedicine.com/portfolio) informed by the patient’s diagnosis or a Stanford Solid Tumor Actionable Mutations Panel (STAMP)(https://stanfordlab.com/content/stanfordlab/en/molecular-pathology/molecular-genetic-pathology.html/). All study components are described in the manuscript. **b** CARE IMPACT pipelines for identifying tumor vulnerabilities. CARE identifies gene expression outliers in each patient’s tumor (hexagon) relative to all other tumors in a large compendium (pan-cancer analysis) and to a subset of the compendium restricted to tumors with similar RNA expression and/or histology (pan-disease analysis). For pan-disease analysis, the focus sample is compared to four cohorts to identify outliers. If an outlier is detected by at least two pan-disease cohorts, it is considered a consensus outlier. Fusion and RNA variant pipelines are also applied to identify expressed mutations and fusions.

The Comparative Analysis of RNA Expression to Improve Pediatric and Young Adult Cancer Treatment (CARE IMPACT) study (Fig. 3a) was conducted collaboratively by the UC Santa Cruz (UCSC) Treehouse Childhood Cancer Initiative and Stanford University School of Medicine. Patients under 30 years of age with a known or suspected recurrent/refractory solid tumor or relapsed leukemia undergoing tumor sampling as part of their standard care were eligible. Patients with a newly diagnosed high-risk cancer for which there was no established standard of care were also included.

Prior to any study procedures, informed consent (from patients over 18 years of age or the patient’s legal guardian for those under 18 years of age) and assent (from patients 7 to 18 years of age) were obtained according to institutional guidelines. Before initiation, this study was approved by the Institutional Review Boards at Stanford University (Human Subject Research Protocol 44179) and the University of California Santa Cruz (HS-FY2024-72). All handling of patient data was performed in accordance with the Declaration of Helsinki.

### RNA sequencing protocol

For each patient, fresh-frozen biopsy or resection samples taken at the time of enrollment or from earlier procedures were sent to Covance by Labcorp (Covance) for RNA-Seq. RNA was extracted with the Qiagen RNEasy kit. A sequencing library was prepared with the Illumina TruSeq Stranded mRNA Library Preparation and sequenced on an Illumina HiSeq 2500 sequencer to obtain 40–50 million reads.

### Patient data transfer

De-identified clinical (Supplementary Data 1) and mutation information (Supplementary Data 6) were extracted from the medical record of each patient at study entry and sent to the UC Santa Cruz (UCSC) Treehouse Childhood Cancer Initiative for analysis. UCSC Treehouse researchers never received direct patient identifiers during the duration of this study. De-identified clinical data was sent to UCSC investigators and secondary Treehouse identifiers (TH34_XXXX_S0X) were generated that could not be linked to direct patient identifiers. De-identified clinical data retrieved from each patient’s medical record included age, sex, race, ethnicity, cancer diagnosis, disease features, and treatment history.

De-identified raw RNA-Seq datasets for Stanford patients were obtained by UCSC from Covance. Covance uploaded patient FASTQ files to UCSC Treehouse’s encrypted Amazon Web Services (AWS) bucket and provided quality metrics. The RNA-Seq files were downloaded from AWS to UCSC Treehouse’s secure servers. RNA-Seq files with associated clinical metadata were managed using REDCap^30^ electronic data capture tools hosted at Treehouse.

### Sequencing data analysis and CARE IMPACT computation pipelines

From June 4, 2018, to September 24, 2020, UCSC Treehouse obtained and processed RNA-Seq datasets for 40 tumor samples from 38 children and young adults. Six samples were not included in the study because they didn’t pass QC checks described later.

The RNA-Seq analysis (https://github.com/UCSC-Treehouse/pipelines) was uniformly performed as described previously^20^, with the following modifications. The most recent docker for the UCSC Treehouse RNA-Seq analysis pipeline was used (docker command: docker pull quay.io/ucsc_cgl/rnaseq-cgl-pipeline:3.3.4-1.12.3)^31^. For this study, the geneBody_coverage.py tool was not run.

As part of the CARE IMPACT analysis, the automated CARE pipeline was employed (https://github.com/UCSC-Treehouse/CARE) to identify clinically relevant oncogenes and oncogenic pathways in each case. For the purposes of this study, clinically relevant genes were designated as genes whose products could be directly or indirectly targeted through the downstream signaling pathway by an approved drug or an investigational agent in any phase of clinical development (Supplementary Data 7). Publicly available Treehouse polyA compendia (https://treehousegenomics.soe.ucsc.edu/public-data/) were used for contextual analysis of each patient (Supplementary Data 8). The CARE pipeline and algorithm compare an RNA-Seq dataset from a focus sample to comparator cohorts selected from the Treehouse compendia and yields two outputs: (1) datasets molecularly similar to the focus sample and (2) genes that are abnormally expressed in the focus sample. Tumors are considered molecularly similar if the Spearman correlation between their expression profiles is above the 95th percentile of all pairwise correlations within the compendium. Abnormally expressed genes are those exceeding the outlier threshold for the comparator cohort. Outlier thresholds are defined using the Tukey outlier detection method ((Interquartile Range)(1.5) + 75% Quartile). For each focus sample dataset, pan-cancer and personalized pan-disease outlier analyses are performed. Pan-cancer outliers are those exceeding the outlier threshold defined by the entire compendium at the time of the analysis (at least 11,368 tumor RNA-Seq profiles from both adult and pediatric patients). Pan-disease outliers are genes with expression exceeding the outlier threshold from at least two of the four personalized pan-disease cohorts: 1) datasets from tumors with the same diagnosis as the focus sample, 2) molecularly similar RNA-Seq datasets (first degree neighbors), 3) first and second degree neighbors (first degree neighbors plus RNA-Seq datasets molecularly similar to them), and 4) datasets from diseases present among the top 6 most correlated datasets (Fig. 3B). Pan-cancer and pan-disease outliers were analyzed for enrichment of downstream pathways and signaling networks containing genes that could be targeted by available therapies.

In addition to the CARE pipeline, variant calling and fusion detection pipelines were run on all RNA-Seq datasets. Together, these pipelines produced a list of clinically informative findings for each focus sample, including gene overexpression outliers, expressed mutations, expressed fusions, and other highly expressed genes.

### CARE IMPACT curation

In addition to the findings identified by the automated pipelines, results were reviewed by human analysts in several steps. Firstly, if no comparator datasets from the same disease were available for the pan-disease analysis described above, a curated cohort of clinically similar tumors was constructed, in consultation with the pediatric oncologist, to replace the “same disease as focus sample” cohort used in the pan-disease analysis (Fig. 3B). For example, for a patient (TH34_1415) diagnosed with undifferentiated sarcoma NOS, we combined multiple types of soft tissue sarcomas. Secondly, after CARE was run, the analyst would review highly expressed genes (95th percentile) that did not reach the threshold for automated reporting as gene expression outliers but might be relevant based on prior knowledge, literature searches, or other genomic information. In this study, findings categorized as “generated using human curation” are those identified using curated similar disease cohorts; those present in only one of four pan-disease cohorts; highly expressed non-outliers implicated by mutation; and mutations and fusion genes.

### RNA-Seq sample quality control metrics

A previously described quality control (QC) framework^32^ was used to ensure sufficient quality of the RNA-Seq data for identifying overexpressed oncogenes and pathways. This method relies on counting MEND reads (Mapped to human genome, Exonic, and Non-Duplicate). Filtering the total pool of reads in an RNA-Seq sample for MEND reads results in a subpopulation of reads that reflect the integrity and quantity of RNA in the sample and indicate whether the data can be used for robust gene expression quantification. Of the 40 RNA-Seq profiles obtained, 34 passed QC and were included in the study. Six RNA-Seq samples failed QC. For two donors, the first datasets produced failed QC, but we were able to include them in the study because a subsequent sample passed QC. Three other donors were excluded from the study because no datasets, initial or subsequent, passed QC. One QC fail sample (TH34_2292_S01) was also included in the study after additional analysis to determine the validity of all the reported outlier genes, because it was the only sample available from that patient. For this sample, the number of measured genes was low, and the majority of sequencing reads (95%) were duplicates. High numbers of duplicates can potentially cause log_2_(TPM + 1) measurements to be inflated. Additional accuracy quantification analysis was run on this sample and outlier analysis repeated after removing all duplicates. For this QC fail sample, only oncogenes with expression greater than 8.5 log_2_(TPM + 1) were selected for further analysis, to account for the high number of duplicate reads. A gene expression value of 8.5 log_2_(TPM + 1) was chosen as the cutoff expression level because it was the 95th percentile of gene expression in the dataset.

### Datasets used for comparative RNA-Seq analysis

Multiple versions of the Treehouse Gene Expression Reference polyA Compendium were used in this manuscript, each composed of RNA-Seq datasets derived from public repositories and our partner clinical sites (Supplementary Data 8). We released new versions as we acquired new RNA-Seq datasets. Due to the reduction in biological signal that can accompany batch effect removal (10.1093/gigascience/giaa117), we used two levels of review to detect potential batch effects. To detect batch effects at the group level, we reviewed a layout of RNA-Seq samples in the compendia based on gene expression similarity and annotated by disease^33^. Instances in which groups of RNA-Seq datasets with the same disease annotations are not adjacent in expression space are reviewed for likely errors. Secondly, at the time of analysis, we review a table of the samples most similar to the patient’s RNA-Seq profile and investigate any with disease or mutation annotations that would not be expected. In this way, we have identified both mistaken annotations and interesting biology^23^. Treehouse’s gene expression compendia are publicly available (https://treehousegenomics.soe.ucsc.edu/public-data/).

### Gene lists used for pan-disease and pan-cancer analysis

For the pan-disease analysis, 58,581 genes from GENCODE Human Release 23 were used. For pan-cancer analysis an expression- and variance-filtered set of GENCODE 23 genes was used, as enumerated in Supplementary Table 1. First, the expression filter drops any gene where 80% or more of the samples have an expression of 0. Second, the variance filter sorts the remaining non-dropped genes and sorts them by the variance of their expression level across the cohort. The 20% of these genes with the lowest variance are dropped regardless of absolute variance.

### Analysis of overexpressed genes

Overexpressed gene lists for each patient RNA-Seq dataset were analyzed for enrichment of pathways and signaling networks containing genes that could be targeted by available therapies (Supplementary Data 7).

We used the Drug Gene Interaction Database (DGIdb)^34^ to identify which overexpressed genes could be targeted by clinically available inhibitors. DGIdb is an open-source project that searches through publications and other curated databases for known or potential interactions between human genes and available inhibitors. To focus our findings on drug targets with known cancer relevance, we set DGIdb to query drug-gene interactions in the following four curated databases: CIViC, Cancer Commons, My Cancer Genome, and My Cancer Genome Clinical Trial. DGIdb does not contain all known drug-gene interactions, nor does it guarantee that any interaction is an appropriate therapeutic intervention. To address these limitations, we conducted additional literature review and consulted published clinical cancer genomic studies. We prioritized studies that considered gene expression information when assessing the druggability of each gene.

We used the Molecular Signatures Database (MSigDB)^35^ to identify significantly overexpressed cancer pathways in each tumor RNA-Seq dataset by conducting gene set overlap analysis, which computes statistically significant pathways between the input gene list of overexpressed genes and the gene sets in the chosen MSigDB collections “Hallmark Gene Sets” and “Canonical Pathways”.

### RNA variant analysis

This section of our data analysis pipeline (https://github.com/UCSC-Treehouse/pipelines) uses BAM files generated from our RNA-Seq analysis pipeline for (1) alignment based variant detection and (2) variant annotation. Variants in a curated list of clinically relevant mutations (Supplementary Data 9) are called using Freebayes^36^ (https://github.com/freebayes/freebayes) version v9.9.2-27-g5d5b8ac, by comparing the specific genomic loci in the reference and patient genomes. The list of variants outputted by Freebayes are annotated using SnpEff version SnpEff 4.3r^37^. This information was used to complement available DNA mutation data. Of note, our list of clinically actionable mutations was updated once during the duration of this registry study. This pipeline has been dockerized and the code is available at https://github.com/UCSC-Treehouse/mini-var-call.

### RNA fusion analysis

This pipeline uses a docker container (https://github.com/UCSC-Treehouse/fusion-for-core) that runs STAR-Fusion^38^ on paired-end FASTQ files and filters the output against a list of known cancer fusion genes (Supplementary Data 10). FusionInspector^39^ is run on the STAR-Fusion output for additional filtering and quantification. The filtering process requires that both fusion partners are in the known cancer fusion gene list. If there are no clinically relevant fusions in the filtered output, a data analyst reviews the unfiltered list for clinically relevant fusions involving promiscuous fusion partners.

### DNA mutation analysis and classification

When adequate tumor tissue was available, a sample was sent for DNA mutation testing at either Foundation Medicine (https://www.foundationmedicine.com/portfolio) (FoundationOne Heme or FoundationOneCDx, as recommended for tumor type) or Stanford’s Solid Tumor Actionable Mutation Panel (STAMP)(https://stanfordlab.com/content/stanfordlab/en/molecular-pathology/molecular-genetic-pathology.html/) (Supplementary Data 6). In one case (TH34_1447_S01), a tumor sample from a different metastatic site and time point in the patient’s cancer progression was sent for testing. Foundation Medicine and STAMP reports provide a list of variants classified as genomic (pathogenic) findings or as variants of uncertain significance (VUS). For reported pathogenic findings they are further classified as actionable if they have therapeutic implications. For actionable variants, a table is provided listing potential therapies including FDA-approved therapies for patient’s tumor type, FDA-approved therapies in other tumor types, and potential clinical trials (Supplementary Data 6). Tumor DNA mutation data was considered clinically useful for a patient if the mutation reports identified an actionable mutation that could be treated with an FDA-approved therapy, or a clinical trial was available at the time the report was generated. One caveat is that what mutation panels consider actionable changes over time with the rapidly developing field of cancer and clinical trials. For example, a NTRK1 variant was not reported as actionable by Foundation Medicine at the time of analysis, however, now could be considered actionable by TRK inhibitors. Of note, variants annotated by the testing site as “equivocal,” meaning the amplification call is not definitive and should be confirmed by a second source, were still considered actionable if an FDA-approved therapy was listed. Additionally, in one case an activating KRAS mutation was listed as actionable with potential FDA-approved therapies listed that are known standard of care for the patient’s disease type, however, the specific mutation was noted to render the patient resistant to therapy. We did not classify this variant as clinically actionable; however, it was still considered clinically useful because it could ultimately impact treatment decisions.

### Clinical genomics tumor board meetings

Upon completion of the CARE IMPACT analysis, summary research reports were sent to the treating oncologist ahead of a Stanford registry study-specific clinical genomics tumor board meeting. The clinical genomics tumor boards were attended by the treating oncologist, additional pediatric oncologists (some of whom were part of the registry study team), genomics scientists, bioinformaticians, data analysts, nurse practitioners, a genetic counselor, and various trainees. This format allowed for rich interdisciplinary discussion of each case. Prior to each session, clinicians were asked to avoid using HIPAA-protected patient identifiers during case discussions to protect patient privacy. The treating physician presented the patient’s history, including past treatment, current medical status, goals of care, and potential therapies being considered. A Treehouse data analyst presented the RNA-Seq data, including specimen quality metrics, gene expression findings, targeted agents identified, and literature supporting or refuting the use of the targeted agent in the patient’s tumor or similar tumors. Available DNA mutation panel results were also presented. Discussion focused on the strength of the analytical findings, the clinical evidence available to support the use of each identified treatment, and how to prioritize each option in the context of other available treatment options. All findings were divided into “Accepted” and “Declined” based on how clinically useful the clinical team perceived them to be. After discussion in the clinical genomics tumor board and any further analysis prompted by the discussion was complete, a final summary report was sent to the treating oncologist including molecular testing results, a TumorMap^33^ visualization of molecularly similar samples, clinically relevant overexpressed genes and pathways, and suggestions for targeted treatments. For patients who received a treatment nominated by the CARE IMPACT analysis, therapeutic benefit was defined as stable or decreasing evidence of disease >6 weeks after initiation of the treatment based on the treating oncologist’s assessment of relevant clinical, pathologic, and imaging studies. Patients with therapeutic benefit were followed for disease and survival outcomes.

### Assessment of clinical utility of CARE IMPACT findings

At a timepoint >6 months from study enrollment, each patient’s medical record was reviewed, and the treating oncologist was interviewed to determine the clinical utility of each CARE IMPACT finding. The clinical utility of each finding was categorized as follows and is displayed in Supplementary Fig. 1:

1. Accepted and prioritized - The targeted agent was FDA-approved, had published phase I safety data in children, and/or the treating oncologist felt the drug should be prioritized over other therapeutic options.
2. Accepted and deferred - The targeted agent was FDA-approved, had published phase I safety data in children, and/or the treating oncologist felt comfortable prescribing the treatment but chose not to do so in favor of another option for which there was more published evidence of efficacy.
3. Declined - The targeted agent was either not FDA-approved, lacked phase I safety data in children, or existing clinical trial evidence suggested a lack of efficacy.

For assessing the patient-level value of CARE IMPACT findings, the finding deemed most promising for each patient by the treating clinician was used for further categorization.

### Determining analysis turnaround time

Turnaround times were calculated for different steps in the clinical registry study workflow (Supplementary Data 2). For each sample different timepoints were collected including date of tumor sample collection, date sample was shipped to Covance for sequencing, date sample was received at Covance, date RNA-Seq files were sent to UCSC Treehouse, date automated Treehouse analysis process completed, date Treehouse hosted an internal mock clinical genomics tumor board meeting, date of final clinical genomics tumor board meeting with UCSC and Stanford. Of note, in some cases a banked sample was used for analysis resulting in a large time from sample collection to being sent to Covance for sequencing.

### Assessment of comparator cohort impact on outlier detection

To determine whether the choice of comparator cohort influenced the outlier analysis in a clinically meaningful way and to compare gene expression outlier approaches used in other precision medicine studies incorporating RNA-Seq^16–18^, we assessed the results of outlier detection with four different pre-defined comparison cohorts: the full compendium (equivalent to CARE pan-cancer analysis, *n* = 12,747), all TCGA datasets (*n* = 9806), data from pediatric patients (age at diagnosis <30 years, *n* = 2814), and data from all cases from a single institution cohort (Stanford, *n* = 110). Of the 12,747 RNA-Seq datasets in the compendium, 9806 (76.9%) are from TCGA; of those, 9440 (96.3%) are adults. Of the 2941 non-TCGA datasets, 96.8% are pediatric (age at diagnosis <30 years). This analysis was based on automated findings using the most recent compendium and CARE IMPACT version; no curation was performed.

## Supplementary information

Supplementary Data 1

Supplementary Data 2

Supplementary Data 3

Supplementary Data 4

Supplementary Data 5

Supplementary Data 6

Supplementary Data 7

Supplementary Data 8

Supplementary Data 9

Supplementary Data 10

Supplementary Information

## Data Availability

Processed RNA sequencing data that support the findings of this study can be accessed here: https://treehousegenomics.soe.ucsc.edu/public-data/. The code for the CARE algorithm used in this manuscript is available via this link https://github.com/UCSC-Treehouse/CARE. The underlying code for data analysis done in this study can be accessed via this link https://github.com/UCSC-Treehouse/CARE_IMPACT_analysis_for_manuscript. Calculations were performed and figures generated with R and RStudio using the following packages: tidyverse, colorspace, cowplot, ggVennDiagram, ggforce, ggrepel, gridExtra, haven, janitor, jsonlite, kableExtra, khroma, knitr, networkD3, RColorBrewer, redcapAPI, UpSetR, webshot.

## Code availability

The code for the CARE algorithm used in this manuscript is available via this link https://github.com/UCSC-Treehouse/CARE. The underlying code for data analysis done in this study can be accessed via this link https://github.com/UCSC-Treehouse/CARE_IMPACT_analysis_for_manuscript. Calculations were performed and figures generated with R and RStudio using the following packages: tidyverse^40^, colorspace^41^, cowplot^42^, ggVennDiagram^43^, ggforce^44^, ggrepel^45^, gridExtra^46^, haven^47^, janitor^48^, jsonlite^49^, kableExtra^50^, khroma^51^, knitr^52^, networkD3^53^, RColorBrewer^54^, redcapAPI^55^, UpSetR^56^, webshot^57^.
