# Supplementary material for: Comparative analysis of RNA expression in a single institution cohort of pediatric cancer patients
Source: NPJ Precis Oncol. 2025 Mar 22;9:81. doi: 10.1038/s41698-025-00852-6 (PMC11928651; doi:10.1038/s41698-025-00852-6)
Supplement: Supplementary file 1 — Supplementary Data 1 [file 41698_2025_852_MOESM1_ESM.pdf]

Supplementary Information

Supplementary Results

Patient cases where Treehouse findings were prioritized and implemented

A) Myoepithelial Carcinoma

A 23-month-old male (TH34_1352_S01) was diagnosed and treated for non-metastatic myoepithelial carcinoma of the liver. After 26 months of ifosfamide/doxorubicin chemotherapy and complete tumor resection he developed bilateral pulmonary metastases. Molecular testing of the pulmonary metastasis found INI-1 deficiency (Supplementary Table 4). RNA-Seq from the same metastatic lung nodule underwent CARE, which found multiple outlier RTKs (FGFR1, FGFR2, PDGFRA), consistent with pan-disease enrichment in FGFR and PDGF pathway signaling, all of which could be targeted with pazopanib (https://www.selleckchem.com/products/pazopanib.html). CARE also identified CCND2 as a pan-disease outlier with pathway support, consistent with the role of SMARCB-1 as a repressor of the cell cycle (Supplementary Table 3). This abnormality is targetable by ribociclib.[1] Therefore, Treehouse analysis nominated both pazopanib and ribociclib for this patient.

After ribociclib treatment, surgical resection and ribociclib maintenance treatment the patient shows no evidence of disease. This case is described in detail in a separate manuscript.

B) Gastrointestinal Stromal Tumor

A 17-year-old young male (TH34_1349_S01/S02) with a germline mutation in *SDHC*, and previous diagnosis of and Hereditary Paraganglioma- Pheochromocytoma Syndrome type 3 (Carney-Strakis), developed recurrent wild-type gastrointestinal stromal tumor (GIST) in the posterior stomach wall with regional nodal and liver metastases nearly 7 years after undergoing wide resection alone. Further tumor progression after 15 months of imatinib therapy prompted complete resection of the gastric mass and enlarged regional lymph nodes and subtotal resection of unresectable liver metastases; pathology confirmed the presence of recurrent GIST with nodal and liver involvement.

RNA-Seq data from the gastric mass and from a liver metastasis were analyzed, and findings in both samples were prioritized as treatment targets, since any treatment based on them could target both sites of the disease. In the gastric sample only, CARE identified *IGF2* as a pan-cancer overexpression outlier with pan-cancer and pan-disease pathway enrichment in the hallmark KRAS signaling pathway. In the liver sample only, *PTCH1* was identified as a pan-cancer and pan-disease overexpression outlier, accompanied by pan-cancer pathway enrichment of the hallmark Hedgehog signaling pathway (Supplementary Table 3).

In both samples, CARE found *KIT* and *ETV1* as pan-cancer and pan-disease overexpression outliers. The receptor tyrosine kinase *KIT* activates the RAS/RAF/MEK pathway,[2] consistent with pan-cancer and pan-disease pathway enrichment in the hallmark KRAS signaling pathway in both samples. This pathway activates transcription factor *ETV1*, leading to cell invasiveness and metastasis as well as transcription of *KIT*.[3] Most GIST tumors harbor an activating *KIT* mutation which makes them sensitive to kinase inhibitors imatinib and sunitinib.[4] This tumor did not have a *KIT* mutation but still displayed overexpression at the RNA level, indicating that *KIT* signaling may be a tumor driver. Several studies have shown benefit of sunitinib treatment for GIST patients with wild-type *KIT,*[5–8] suggesting that *KIT* RNA overexpression may be another predictive biomarker of response to KIT inhibitors, especially in the setting of wild-type *KIT*.

The patient’s therapy was switched to sunitinib. This line of treatment was consistent with the Treehouse analysis, although sunitinib is also standard of care second-line therapy for GIST[9,10]. MRI imaging 37 months after starting sunitinib showed stable disease.

C) Osteosarcoma

In one case (TH34_1456_S02) a therapy was chosen which targets a Treehouse analysis finding, but this therapy was chosen for a different reason. A 13-year-old male patient had been diagnosed with osteosarcoma metastatic to the lung. CARE analysis of RNA-Seq data from a lung metastasis identified *KDR/VEGFR2* as a pan-cancer and pan-disease up-outlier with pathway support (Supplementary Table 3), consistent with *VEGFA* amplification found by STAMP panel (Supplementary Table 4). The clinician chose treatment with cabozantinib, an RTK inhibitor, because it has relatively high rates of disease control in recurrent osteosarcoma and is well-tolerated.[11] The patient had a partial response after 2 months and continued stable disease for an additional 12 months thereafter. Although this treatment was chosen based on prior evidence of efficacy in osteosarcoma, cabozantinib is an RTK inhibitor with high affinity for *KDR/VEGFR2*, and the patient's response may be related to the outlier expression of *KDR/VEGFR2*.

D) Posterior Fossa Ependymoma

A 2-year-old female (TH34_1381_S01) with posterior fossa anaplastic ependymoma with loss of H3K27 trimethylation by immunohistochemistry underwent resection of a frontal lobe metastasis after past treatment with surgery and radiotherapy for the primary tumor, a local recurrence, and a metachronous metastatic recurrence in the same area of the frontal lobe. Loss of H3K27 trimethylation is a hallmark of posterior fossa ependymoma group A (PFA), and is associated with very poor prognosis.[12,13] Foundation Medicine reported amplification of *IKBKE, MCL1,* and *NTRK1* in the tumor (Supplementary Table 4). Additional CyberKnife radiotherapy and 10 cycles of oral etoposide were given. Two months after discontinuing etoposide, disease progression was observed in the posterior fossa.

CARE analysis of the frontal lobe metastasis identified *VEGFA* as a pan-cancer and pan-disease overexpression outlier supported by pan-disease enrichment of the Hallmark Hypoxia pathway (Supplementary Table 3). *ERBB2* and *PARP1* were also identified as pan-disease up-outliers. *ERBB2* (*NEU*, *HER2*) encodes a member of the epidermal growth factor (EGF) receptor family of receptor tyrosine kinases. A multicenter Phase II clinical trial of *HER2* inhibitor lapatinib showed modest activity against brain tumor metastases in *HER2*-positive breast cancer patients previously treated with trastuzumab.[14] In addition, a single group phase II study showed that the combination of lapatinib and capecitabine is active as first-line treatment of brain metastases from *HER2*-positive breast cancer.[15]

The patient was enrolled on a clinical trial of oral neratinib, a HER2 inhibitor. An MRI after 2 cycles showed possible pseudo progression, but an MRI after 3 cycles showed definite tumor progression, so the patient was removed from the study and the family elected no further cancer-directed therapy.

E) Embryonal Rhabdomyosarcoma

A 4-year-old female (TH34_2351_S01) with left neck embryonal rhabdomyosarcoma metastatic to multiple bones was treated with multiagent chemotherapy, delayed resection of the primary tumor and radiotherapy. A left neck nodal recurrence 4 years after therapy completion was treated with vinorelbine, cyclophosphamide, and temsirolimus, surgery, and radiotherapy. Three months after therapy completion, a nasopharyngeal recurrence was identified and treated with vincristine, irinotecan, and temozolomide. A subsequent biopsy of the nasopharyngeal recurrence was sent for RNA sequencing and then to Treehouse for analysis.

While awaiting CARE results, the patient was started on pazopanib, because it is FDA-approved for recurrent soft tissue sarcomas in adults and some preclinical data suggests a benefit in pediatric patients.[16,17] CARE found only one targetable overexpression outlier: *HMOX1* was a pan-disease outlier with pathway support in the PID HIF1 TF Pathway and Hallmark P53 pathway (Supplementary Table 3, Figure 4). Additionally, Treehouse RNA variant calling identified an activating *NRAS* G12D mutation (alt/ref 255/103). Because *NRAS* is an upstream activator of *MAP2K1/MEK*, we investigated the expression of *MAP2K1/MEK* and found that it had expression above the 95th percentile in the Treehouse compendium but was not an overexpression outlier.

In the absence of clinical improvement on pazopanib, the patient was switched to trametinib, a *MEK* inhibitor, but 20 days later trametinib was stopped due to symptomatic local progression in the face, for which the patient received palliative RT. The lack of response to trametinib may be attributable to the fact that our assay focuses on targetable overexpression outliers, but *MEK* did not have outlier expression.

Patient cases where Treehouse findings were prioritized but not implemented

A 16-year-old male (TH34_1240_S01) with pulmonary metastatic Ewing sarcoma developed progressive pulmonary metastases along with nodal and bony metastases while receiving up-front standard chemotherapy and after undergoing delayed wide resection of the primary left scapular tumor. Further disease progression in the lungs and lymph nodes, along with soft tissue and liver metastases were observed despite treatment with several salvage chemotherapy regimens. Following Treehouse findings that MYC was a pan-disease overexpression outlier with pathway support (Supplementary Table 3), the patient would have been placed on a phase I clinical trial of a MYC-targeted drug, but the patient was too ill to qualify for the study and he died without receiving any further systemic therapy.

In a second case, a male patient (TH34_1379_S01) was diagnosed with hepatoblastoma/transitional cell carcinoma of the liver at 4 years of age, with recurrence at 10 years of age. The Treehouse analysis identified *FGFR1* as a pan-disease overexpression outlier, supported by pathway enrichment in the Hallmark Angiogenesis Pathway (Supplementary Table 3). FGFR proteins can be targeted by pazopanib, an RTK inhibitor. The patient received a cycle of docetaxel and the clinician planned to give a second cycle of docetaxel along with pazopanib, but the patient died before receiving further therapy.

A 15-year-old male (TH34_1380_S01) was diagnosed with embryonal rhabdomyosarcoma with multiple metastases. Several genomic alterations were noted by Foundation Medicine and STAMP (Supplementary Table 4). CARE identified *NOTCH3* (therapy: tarextumab) as a pan-cancer and pan-disease up-outlier with pathway support, and *IGF2* as a pan-cancer up-outlier (therapy: ganitumab)(Supplementary Table 3). The clinician elected to give pazopanib instead because it is FDA-approved for recurrent sarcomas in adults and there is some evidence of clinical utility in rhabdomyosarcoma. However, disease progression was observed after 2 cycles and palliative RT was given due to pain and spinal cord compression. During RT, the clinician initiated a compassionate use IND for the NOTCH inhibitor nirogacestat. However, oral etoposide was subsequently started since the patient’s mother did not want to wait any longer for systemic therapy, and etoposide is known to be an active agent in rhabdomyosarcoma. He was subsequently transitioned to hospice care.

A male patient (TH34_1455_S01) with relapsed ependymoma would have been placed on a neratinib phase II study following Treehouse findings that *ERBB2* was a pan-disease overexpression outlier (Supplementary Table 3), but he was ineligible for the trial due to elevated liver function tests.

A 22-year-old male patient (TH34_2292_S01) was diagnosed with undifferentiated round cell sarcoma with brain metastases. Foundation Medicine reported CIC rearrangement, exon 20 (Supplementary Table 4). Prior to CARE, the patient was started on trabectedin because there is some evidence that it may be useful in CIC-rearranged soft tissue sarcomas. CARE identified *FLT4* (therapy: pazopanib) as a pan-cancer up-outlier with pathway support, and *VEGFA* (therapy: bevacizumab) as a pan-cancer up-outlier with pathway support (Supplementary Table 3). The clinician was interested in the Treehouse findings, but the patient was not able to get either therapy because he was unable to tolerate any oral medications (pazopanib) and he did not get bevacizumab because he was already on trabectedin by the time the Treehouse results were available. The clinician would have considered bevacizumab therapy after trabectedin, but the patient was never well enough to receive further therapy and died after resection of the brain metastases.

Patient with a rare tumor with no standard therapies

A 16-year-old young woman who presented with severe anemia (hemoglobin 4.1 g/dL) underwent partial excision of a necrotic, pedunculated mass extending from the uterus through the cervical os; pathology showed high-grade endometrial stromal sarcoma with YWHAE-NUTM2 gene fusion. Laparoscopic total hysterectomy and bilateral pelvic lymph node dissection confirmed tumor extension to the parametrium and lymph node metastases. Computed tomography imaging of the chest showed 3 small, solid bilateral pulmonary nodules suggestive of metastases. In the absence of a clear standard of care, particularly in a pediatric patient, dose-intensive ifosfamide/doxorubicin chemotherapy, and external beam radiotherapy 48.6 Gy to the whole pelvis were given. Due to the anticipated very poor prognosis, a portion of the primary tumor resection specimen was sent to Treehouse for CARE analysis.

CARE analysis of RNA-Seq data from a primary tumor resection identified the estrogen receptor *ESR1* as a pan-cancer and pan-disease overexpression outlier (Supplementary Table 3). Downstream targets of ESR1, CCND1 and CDK4,[18] were found to be highly expressed in the sample and CDK4/6 inhibitor palbociclib was recommended as a potential therapy. CARE also identified elevated expression of *MAPK11*, which is known to lead to increases in cell survival,[19] and *ABCB1*, which was identified as a pan-disease overexpression outlier. Based on *ABCB1* being known to increase resistance to chemotherapy,[20,21] regorafenib and ulixertinib were recommended for their known ability to overcome *ABCB1*-mediated chemotherapeutic drug resistance.[22,23] In this case, the clinician chose to defer all Treehouse findings while the patient received standard chemotherapy and radiotherapy. The patient currently remains free of disease recurrence 22 months from therapy completion.

Data Acknowledgment

Use of data available in public repositories allowed us to increase the number of pediatric cancer patients and pediatric cancer types included in our reference compendium; we gratefully acknowledge the data providers listed in Supplementary Table 5 in the manner they have specified.

Use of data from partners and available in public repositories allowed us to increase the number of pediatric cancer patients and pediatric cancer types included in our reference compendium, and we gratefully acknowledge the following data providers in the manner they have specified:

Tumor Compendium v11 Public PolyA (April 2020)
This research was conducted using data made available by the following institutions:

British Columbia Cancer Agency
The Children's Brain Tumor Tissue Consortium
The Hospital for Sick Children
Stanford University
University of Calgary
University of California, San Francisco

International Cancer Genome Consortium. The following ICGC datasets were used in this work: EGAD00001000158, EGAD00001001620, EGAD00001000328, EGAD00001000648, EGAD00001000617, EGAD00001000826 and EGAD00001000356.
St. Jude Children's Research Hospital – Washington University Pediatric Cancer Genome Project; the following datasets were obtained via EGA (https://www.ebi.ac.uk/ega/) and were used by permission: EGAD00001001098: Andersson AK, Ma J, Wang J, et al. The landscape of somatic mutations in infant MLL-rearranged acute lymphoblastic leukemias. Nat Genet. 2015;47(4):330-7. (PMC4553269). EGAD00001001666: Qaddoumi I, Orisme W, Wen J, et al. Genetic alterations in uncommon low-grade neuroepithelial tumors: BRAF, FGFR1, and MYB mutations occur at high frequency and align with morphology. Acta Neuropathol. 2016;131(6):833-45. (PMID 26810070). EGAD00001002680: Pinto EM, Chen X, Easton J, et al. Genomic landscape of paediatric adrenocortical tumours. Nat Commun. 2015;6:6302. (PMC4352712). Dataset SJC-DS-1001 was accessed and processed with permission from St. Jude Cloud (https://www.stjude.cloud) – a publicly accessible pediatric genomic data resource requiring approval for controlled data access:

Chen, X, et al. Targeting oxidative stress in embryonal rhabdomyosarcoma. Cancer Cell. 2013 Dec 9;24(6):710-24.
Gruber TA, et al. An Inv(16)(p13.3q24.3)-encoded CBFA2T3-GLIS2 fusion protein defines an aggressive subtype of pediatric acute megakaryoblastic leukemia. Cancer Cell. 2012; 22(5):683-697.
Holmfeldt L, et al. The genetic landscape of hypodiploid acute lymphoblastic leukemia. Nat. Genet. 2013.
Robinson G, et al. Novel mutations target distinct subgroups of medulloblastoma. Nature. 2012; 488(7409):43-48.
Zhang J, et al. Whole-genome sequencing identifies genetic alterations in pediatric low-grade gliomas. Nat. Genet. 2013; 45(6):602-12.
Zhang J, et al. A novel retinoblastoma therapy from genomic and epigenetic analyses. Nature. 2012; 481(7381):329-334.
Zhang J, et al. The genetic basis of early T-cell precursor acute lymphoblastic leukaemia. Nature. 2012; 481(7380):157-163.
Provider(s) of the data under accession phs000178.v10.p8 at dbGap. The Cancer Genome Atlas Research Network, National Cancer Institute and National Human Genome Research Institute, Bethesda, MD, USA. The results published here are in part based upon data generated by The Cancer Genome Atlas managed by the NCI and NHGRI. Information about TCGA can be found at http://cancergenome.nih.gov/.
Provider(s) of the data under accession phs000178.v10.p8 at dbGap: The Therapeutically Applicable Research to Generate Effective Treatments (TARGET) initiative managed by the NCI. The data used for this analysis are available at dbGap under accession phs000218. Information about TARGET can be found at http://ocg.cancer.gov/programs/target.

Children's Oncology Group (COG; Tissues for TARGET are collected as part of COG clinical and biological protocols)
Peter C. Adamson, MD. Children's Hospital of Philadelphia, Philadelphia, PA, USA
Principal Investigator (ALL Project Team)
Stephen P. Hunger, MD. University of Colorado Cancer Center, Denver, CO, USA
Principal Investigators (AML Project Team)
Soheil Meshinchi, MD, PhD. Fred Hutchinson Cancer Research Center, Seattle, WA, USA
Robert Arceci, MD, PhD. Children's Hospital, Phoenix, AZ, USA
Principal Investigator (NBL Project Team)
John M. Maris, MD. Children's Hospital of Philadelphia, Philadelphia, PA, USA
Robert Seeger, MD. Children's Hospital of Los Angeles, Los Angeles, CA, USA
Javed Khan, MD. National Cancer Institute, National Institutes of Health, Bethesda, MD, USA
Principal Investigator (OS Project Team)
Ching Lau, MD, PhD. Texas Children's Hospital, Houston, TX, USA
Paul Meltzer, MD, PhD. National Cancer Institute, National Institutes of Health, Bethesda, MD, USA
Principal Investigator (Kidney Project Teams - WT, CCSK, RT)
Elizabeth J. Perlman, MD. Ann and Robert H. Lurie Children's Hospital of Chicago, Chicago, IL, USA
Principal Investigator (Cell Lines and Xenografts - PPTP)
Peter Houghton, PhD. The Research Institute at Nationwide Children's Hospital, Columbus, OH, USA

Provider(s) of the data under accession phs000699.v1.p1 at dbGap: Todd Golub. Dana Farber Cancer Institute, Boston, MA, USA. This work was conducted as part of the Slim Initiative for Genomic Medicine in the Americas (SIGMA), a project funded by the Carlos Slim Health Institute in Mexico.

Provider(s) of the data under accession phs000900.v1.p1 at dbGap: Michelle Monje, Stanford University, Stanford, CA, USA. We thank the many patients and families who selflessly contributed to this study through tissue donations from surgery or autopsy and Amar Gajjar, for his guidance and vision throughout this study. We also thank Darren Hargrave, James Olson and Sarah Leary for selection of V.2 chemical screen agents. We are grateful for the critical questions and comments by Simone Cheetham and Nadim Nsouli. We also acknowledge important comments by other DIPG Preclinical Consortium member Oren Becher. We thank Gerald Grant for assistance in developing rodent CED techniques. Short read sequencing was performed by the OHSU Massively Parallel Sequencing Shared Resource. The first paper using this data was: Grasso CS, Tang Y, Truffaux N, et al. Functionally defined therapeutic targets in diffuse intrinsic pontine glioma. Nat Med. 2015;21(6):555-9. (PMID 25939062).

Provider(s) of the data under accession phs000720.v2.p1 at dbGap: Javed Khan, MD. National Institutes of Health, Bethesda, MD, USA. The authors thank the Children's Oncology Group Soft Tissue Sarcoma Committee and the BioPathology Center, for their careful collection of clinical samples. This research was supported by the Intramural Research Program of the National Institute of Health and National Cancer Institute.

Provider(s) of the data under accession phs000768.v2.p1 at dbGap. Javed Khan, MD. National Institutes of Health, Bethesda, MD, USA. This research was supported by the Intramural Research Program of the National Institute of Health and National Cancer Institute. The datasets have been accessed through the NIH database for Genotypes and Phenotypes (dbGaP) under accession # phs000768.v1.p1.

Provider(s) of the data under accession phs000673.v2.p1 at dbGap. Arul Chinnaiyan, MD PhD. Michigan Center for Translational Pathology, University of Michigan, MI, USA. The results published here are in whole or part based upon data generated by the Clinical Sequencing Exploratory Research (CSER) consortium established by the NHGRI. Funding support was provided through cooperative agreements with the NHGRI and NCI through grant numbers U01 HG006508 (Exploring Cancer Medicine for Sarcoma and Rare Cancers). Information about CSER and the investigators and institutions who comprise the CSER consortium can be found at http://www.genome.gov/27546194

Provider(s) of the data under accession SRP040454 at SRA. Dataset was made available by Memorial Sloan Kettering Cancer Center.

Supplementary Figures

Supplementary Figure 1. Clinical utility of CARE IMPACT findings in 33 patients

The findings are arranged on the y-axis and grouped into “Accepted” and “Declined” categories as described in Supplemental Methods. The details of the findings are described in Supplemental Results.

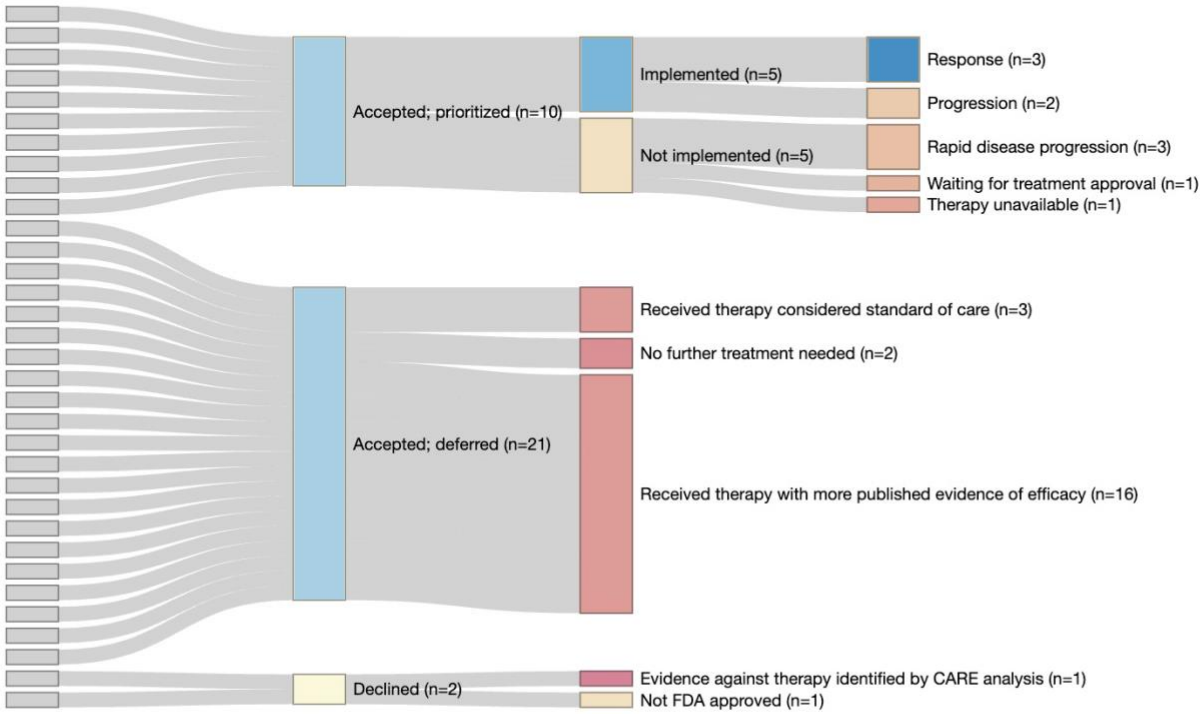

Supplementary Figure 2. Outliers detected by different comparative cohorts

The outlier gene is shown on the y-axis, while patient RNA-Seq sample ID's are shown on the x-axis. The colors indicate the comparator cohort used to identify the outlier gene.

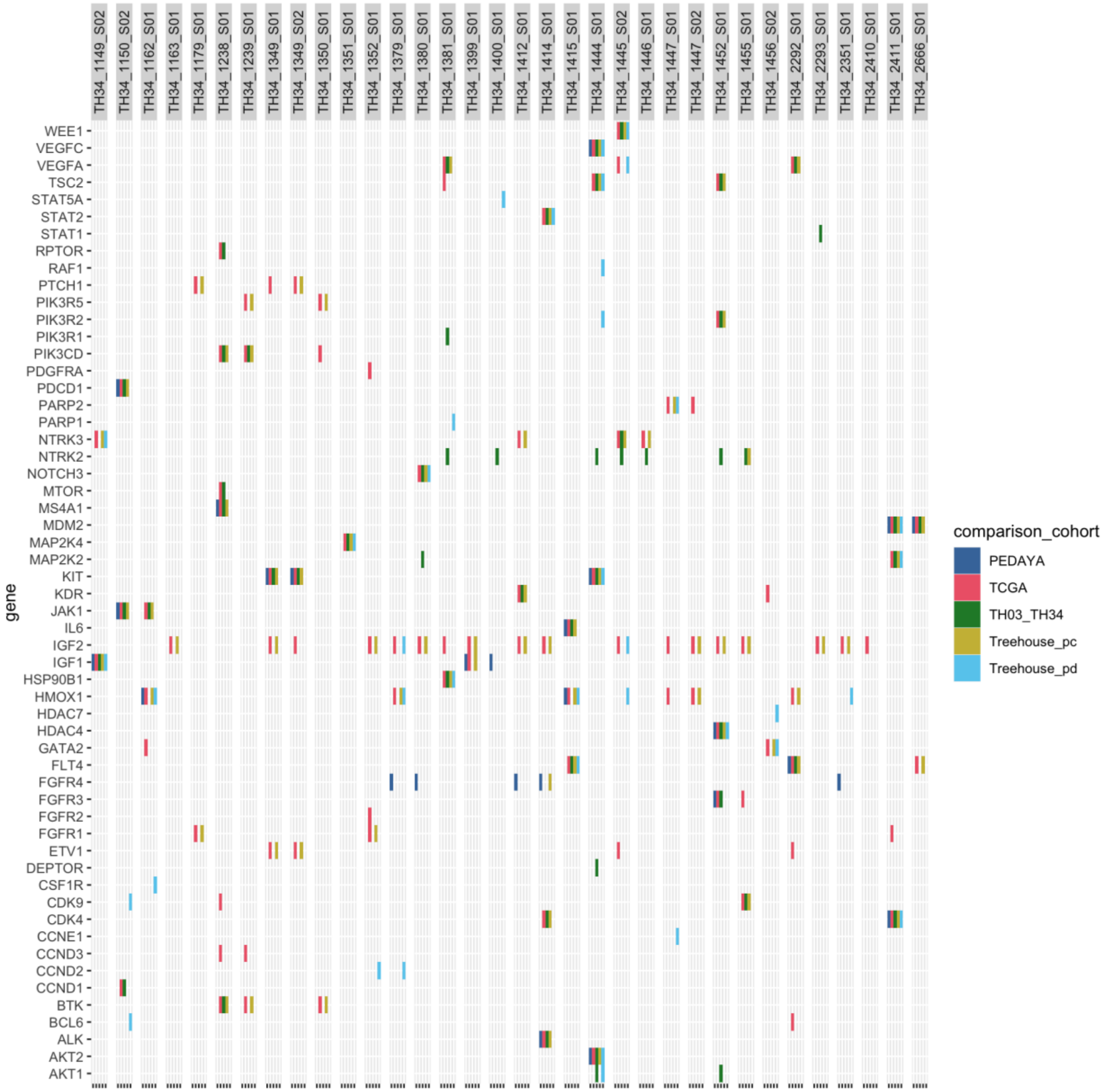

Supplementary Figure 3. Outliers detected relative to cohorts

The total number of outliers detected relative to each cohort is displayed on the horizontal bars on the left of the cohort name. Vertical bars depict the number of outliers detected relative to each combination of cohorts indicated by black points. The largest set consists of 27 outliers detected relative to both the Treehouse pan-cancer cohort and TCGA. Combinations yielding no outliers are omitted.

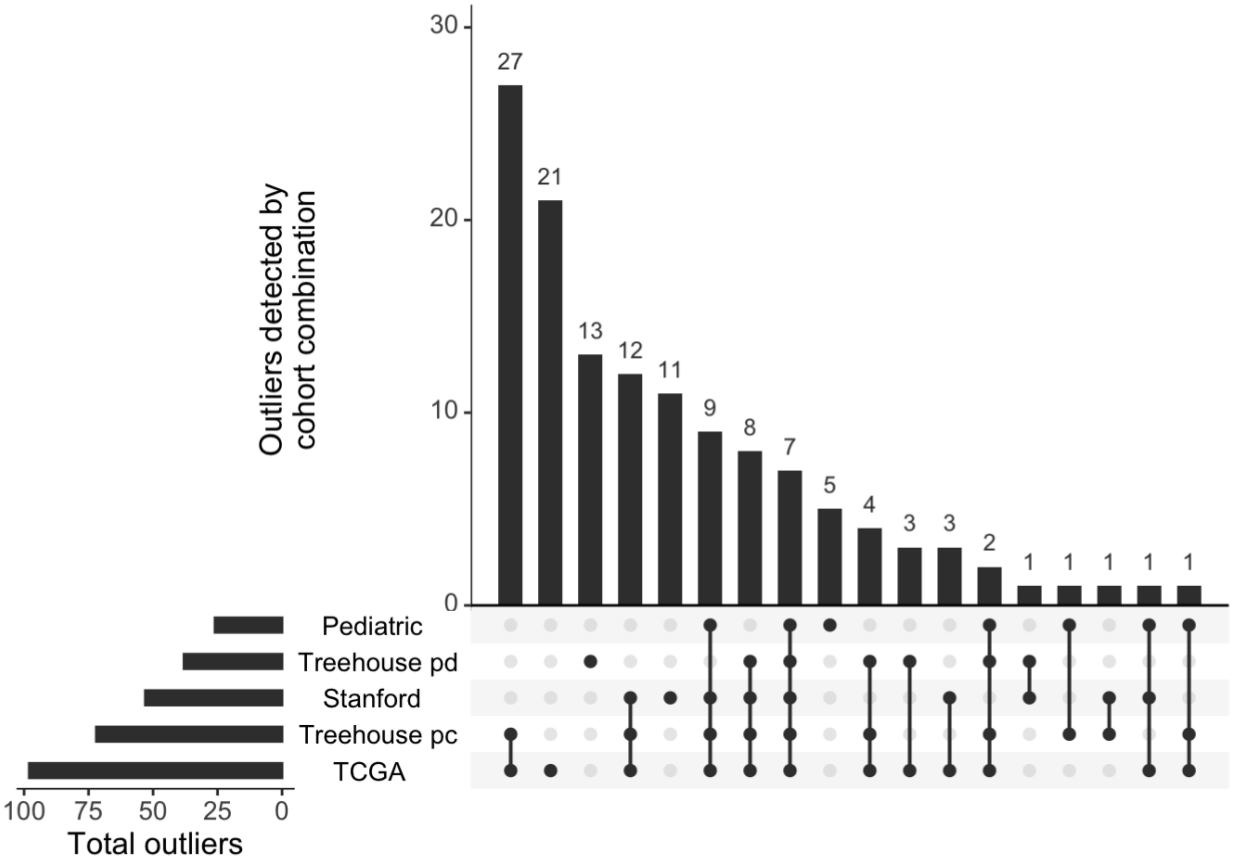

Supplementary Tables

Supplementary Table 1. Size of filtered gene lists for pan-cancer analysis

This table enumerates genes filtered from the 58,581 genes from GENCODE Human Release 23 for low expression and high variance of expression used in pan-cancer analysis for different compendia versions.

**Treehouse PolyA Compendia Version**	**Number of Expression Filtered Genes (N)**	**Number of Variance Filtered Genes (n)**	**Number of Total Filtered Out Genes (N+n)**	**Number of Genes After Filtering (58581 - (N+n))**
v7	24716	6773	31489	27092
v8	24676	6781	31457	27124
v9	24726	6771	31497	27084
v10	27222	6806	24553	31359
v11	24421	6832	31253	27328

Supplementary Data

Supplementary Data 1. Patient demographics and clinical information

Clinical characteristics for each patient enrolled in the CARE IMPACT study.

Supplementary Data 2. Key registry timepoints per sample

For each sample different timepoints were collected including date of tumor sample collection, date sample was shipped to Covance for sequencing, date sample was received at Covance, date RNA-Seq files were sent to UCSC Treehouse, date automated Treehouse analysis process completed, date Treehouse hosted an internal mock clinical genomics tumor board meeting, date of final clinical genomics tumor board meeting with UCSC and Stanford.

Supplementary Data 3. CARE IMPACT findings by sample

Table of CARE IMPACT findings nominated for each patient's sample. Tumor vulnerability category refers to the category a gene falls under in terms of its function. Therapies recommended to clinicians based on the CARE IMPACT finding are included. For each nominated therapy, the clinical utility categorization determined by the clinician as well as the reason for that categorization is provided (Clinical_Prioritization). CARE IMPACT finding source indicates whether the finding was identified by the CARE IMPACT pipelines as gene overexpression outliers (pan-cancer vs pan-disease), expressed mutations, expressed fusions, or other highly expressed genes. Pan-disease findings are further categorized by the cohorts used and the number of pan-disease cohorts used to detect them. Consensus outliers were defined as those identified by at least two of the predefined pan-disease cohorts as described in the methods. Canonical cohorts include the four personalized pan-disease cohorts: 1) datasets from tumors with the same diagnosis as the focus sample, 2) molecularly similar RNA-Seq datasets (first degree neighbors), 3) first and second degree neighbors (first degree neighbors plus RNA-Seq datasets molecularly similar to them), and 4) datasets from diseases present among the top 6 most correlated datasets. Curated cohorts are those that involved action from a human analyst. Details on curation are provided in the notes column. The compendium used for the CARE IMPACT analysis is included.

Supplementary Data 4. Outliers detected for each dataset

Table of outliers detected in each sample. The comparison cohort used to detect the outlier gene is indicated, as well as whether there was pathway support for the outlier expression.

Supplementary Data 5. Cohort-specific features of expression distributions for TCGA-only outlier genes

Table of genes for which the interquartile ranges (IQR) calculated for the PEDAYA (pediatric and young adult) and TCGA were different. The IQR calculated for each gene in each cohort is provided, along with the median expression value for the cohort and the change in IQR for each cohort relative to each other.

Supplementary Data 6. DNA variant calls per patient sample

Table of pathogenic variants detected in each patient's sample by DNA mutation testing done at either Foundation Medicine or by the Stanford's Solid Tumor Actionable Mutation Panel (STAMP). Reported pathogenic findings they are classified as actionable if they have therapeutic implications. For each variant potential therapies including FDA-approved therapies for patient's tumor type, FDA-approved therapies in other tumor types, and potential clinical trials are listed. Variants labeled as "equivocal," indicate that the amplification call is not definitive and should be confirmed by a second source. Samples for which DNA mutation testing was not done and the reason why are included at the bottom of the table.

Supplementary Data 7. Directly and indirectly actionable genes used to prioritize gene expression outlier findings

Table of genes designated as clinically relevant by UCSC Treehouse. Category refers to the category a gene falls under in terms of its function. Druggability refers to whether a drug can be directly targeted or indirectly targeted through the downstream signaling pathway. Known drugs are listed based on whether they were FDA approved or an investigational agent in any phase of clinical development at the time of the study.

Supplementary Data 8. Published repository datasets included in the Treehouse Compendia

Description of data included in Treehouse public polyA Gene Expression data Compendia and number of samples in each compendia version. Includes name of datasets, accession IDs, project names, repository source, diseases included in datasets, and number of samples from each dataset included in each compendia version. Repository samples are those that were downloaded from publicly available data repositories. Partner samples are those that were received from clinical partners (i.e., Stanford). The release year for each compendia version is given (i.e., May 2018).

Supplementary Data 9. Variants assessed in RNA-Seq data

List of clinically relevant variants curated by the UCSC Treehouse team for the RNA variant analysis pipeline. Includes chromosome location, start position, stop position, and detectable variant.

Supplementary Data 10. Known cancer fusion genes

List of known cancer fusion genes curated by the UCSC Treehouse team for the RNA fusion analysis pipeline.

Supplementary References

1. Kohashi, K. & Oda, Y. Oncogenic roles of SMARCB1/INI1 and its deficient tumors. *Cancer Sci* **108**, 547–552 (2017).
2. R�nnstrand, L. Signal transduction via the stem cell factor receptor/c-Kit. *CMLS, Cell. Mol. Life Sci.* **61**, 2535–2548 (2004).
3. Chi, P. *et al.* ETV1 is a lineage survival factor that cooperates with KIT in gastrointestinal stromal tumours. *Nature* **467**, 849–853 (2010).
4. Fletcher, J. A. & Rubin, B. P. KIT Mutations in GIST. *Current Opinion in Genetics & Development* **17**, 3–7 (2007).
5. Heinrich, M. C. *et al.* Primary and Secondary Kinase Genotypes Correlate With the Biological and Clinical Activity of Sunitinib in Imatinib-Resistant Gastrointestinal Stromal Tumor. *JCO* **26**, 5352–5359 (2008).
6. Pantaleo, M. A. *et al.* Analysis of all subunits, SDHA, SDHB, SDHC, SDHD, of the succinate dehydrogenase complex in KIT/PDGFRA wild-type GIST. *Eur J Hum Genet* **22**, 32–39 (2014).
7. Rutkowski, P., Magnan, H., Chou, A. J. & Benson, C. Treatment of gastrointestinal stromal tumours in paediatric and young adult patients with sunitinib: a multicentre case series. *BMC Cancer* **17**, 717 (2017).
8. Verschuur, A. C. *et al.* Sunitinib in pediatric patients with advanced gastrointestinal stromal tumor: results from a phase I/II trial. *Cancer Chemother Pharmacol* **84**, 41–50 (2019).
9. George, S. *et al.* Clinical evaluation of continuous daily dosing of sunitinib malate in patients with advanced gastrointestinal stromal tumour after imatinib failure. *European Journal of Cancer* **45**, 1959–1968 (2009).
10. Janeway, K. A. *et al.* Sunitinib treatment in pediatric patients with advanced GIST following failure of imatinib. *Pediatr. Blood Cancer* **52**, 767–771 (2009).
11. Italiano, A. *et al.* Cabozantinib in patients with advanced Ewing sarcoma or osteosarcoma (CABONE): a multicentre, single-arm, phase 2 trial. *The Lancet Oncology* **21**, 446–455 (2020).
12. Bayliss, J. *et al.* Lowered H3K27me3 and DNA hypomethylation define poorly prognostic pediatric posterior fossa ependymomas. *Sci. Transl. Med.* **8**, (2016).

13. Zapotocky, M. *et al.* Survival and functional outcomes of molecularly defined childhood posterior fossa ependymoma: Cure at a cost. *Cancer* **125**, 1867–1876 (2019).

14. Lin, N. U. *et al.* Multicenter Phase II Study of Lapatinib in Patients with Brain Metastases from HER2-Positive Breast Cancer. *Clin Cancer Res* **15**, 1452–1459 (2009).

15. Bachelot, T. *et al.* Lapatinib plus capecitabine in patients with previously untreated brain metastases from HER2-positive metastatic breast cancer (LANDSCAPE): a single-group phase 2 study. *The Lancet Oncology* **14**, 64–71 (2013).

16. Glade Bender, J. L. *et al.* Phase I Pharmacokinetic and Pharmacodynamic Study of Pazopanib in Children With Soft Tissue Sarcoma and Other Refractory Solid Tumors: A Children's Oncology Group Phase I Consortium Report. *JCO* **31**, 3034–3043 (2013).

17. Yoo, K. H. *et al.* Efficacy of pazopanib monotherapy in patients who had been heavily pretreated for metastatic soft tissue sarcoma: a retrospective case series. *BMC Cancer* **15**, 154 (2015).

18. Oza, A. & Ma, C. X. New Insights in Estrogen Receptor (ER) Biology and Implications for Treatment. *Curr Breast Cancer Rep* **9**, 13–25 (2017).

19. Ferrari, G. *et al.* TGF-β1 Induces Endothelial Cell Apoptosis by Shifting VEGF Activation of p38MAPK from the Prosurvival p38β to Proapoptotic p38α. *Molecular Cancer Research* **10**, 605–614 (2012).

20. Abd El-Aziz, Y. S., Spillane, A. J., Jansson, P. J. & Sahni, S. Role of ABCB1 in mediating chemoresistance of triple-negative breast cancers. *Bioscience Reports* **41**, BSR20204092 (2021).

21. Katayama, R. *et al.* P-glycoprotein Mediates Ceritinib Resistance in Anaplastic Lymphoma Kinase-rearranged Non-small Cell Lung Cancer. *EBioMedicine* **3**, 54–66 (2016).

22. Wang, Y.-J. *et al.* Regorafenib overcomes chemotherapeutic multidrug resistance mediated by ABCB1 transporter in colorectal cancer: In vitro and in vivo study. *Cancer Letters* **396**, 145–154 (2017).

23. Ji, N. *et al.* Ulixertinib (BVD-523) antagonizes ABCB1- and ABCG2-mediated chemotherapeutic drug resistance. *Biochemical Pharmacology* **158**, 274–285 (2018).

24. Vaske, O. M. *et al.* Comparative Tumor RNA Sequencing Analysis for Difficult-to-Treat Pediatric and Young Adult Patients With Cancer. *JAMA Netw Open* **2**, e1913968 (2019).
